# The Role of Aspect During Deverbal Word Processing in Greek

**DOI:** 10.1007/s10936-024-10112-6

**Published:** 2025-01-11

**Authors:** Eleni Tsaprouni, Christina Manouilidou

**Affiliations:** Department of Comparative and General Linguistics, Faculty of Arts, University of Ljubljana, Ljubljana, Slovenia

**Keywords:** Aspect, Morphological processing, Greek, Deverbal formations

## Abstract

Deverbal formations in Greek, e.g. *miˈrazo* ‘to distribute’ *< ˈmirazma* ‘distributing’ are considered morphologically complex lexical items. Previous psycholinguistic studies in Greek and English already highlighted the importance of lexical category and argument structure of the base verb in the processing of deverbal pseudowords violating constraints pertaining to these properties. A similar study in Slovenian brought into light the role of aspectual properties of the base verb during deverbal word processing. The present study revisits the role of aspect in morphological processing of deverbal word formations by looking at Greek. To this end, an offline acceptability judgement task and an online lexical decision task were conducted using different types of pseudowords, violating derivational rules. Results provide evidence that aspect affects deverbal pseudoword processing in Greek. Aspectual rules have a distinct role in relation to categorial and argument structure ones for the suffix that creates formations with unambiguous, eventive readings and which has clear event implications but not for other suffixes. The current study extends the literature to include the role of this feature in the processing of deverbal formations.

## Introduction

Morphological processing of complex words has always been an important topic of discussion in psycholinguistics. Taft & Forster’s (1975) decomposition model was the first model accounting for the processing of complex words suggesting that during complex word recognition, affixed words are decomposed into their constituent morphemes, i.e., stem + affix. Then, the lexical representation of the stem is searched for. If the stem is successfully accessed, the full form is retrieved. In general, a significant number of subsequent studies (Forster, 1981, 1987; Murray & Forster, 2004; Taft, 1979, 2004, 2023) support the idea that affixes – irrespective of whether they are prefixes or suffixes, inflectional or derivational, bound or free – are recognized and processed by a single, early, form-based, morphological parsing mechanism. This mechanism operates in a serial way. At an initial stage, the complex word is decomposed into its constituent morphemes, and at a later stage, the stored lexical entries of these morphemes are activated. The activation of the stored lexical entries constitutes a prerequisite for the constituent morphemes to be able to combine with each other, in order to form an interpretable complex word.

Meanwhile, Schreuder & Baayen’s (1995) Interactive Activation Race Model is a hybrid model, according to which full forms and decompositional routes interact with each other in order to converge the meaning of a complex lexical item. Particularly, Schreuder and Baayen (1995) proposed a model, according to which processing of complex words is divided into two main stages, a pre- and a post-decomposition stage, with the latter consisting of three distinct sub-stages. This so-called meta-model of morphological processing is considered to provide insights about the processing of complex words in both modalities, namely visual and auditory. The three distinct processes involved in the post-decomposition stage are: *lexeme lookup*, *(syntactic) licensing* and *(semantic) composition*.

*Lexeme lookup* is the process during which the constituent morphemes of the complex word are retrieved from the mental lexicon, and their lexical features are activated. *Syntactic licensing* checks whether these morphemes and their representations fulfill the prerequisites of their subcategorization properties in order to fit with each other. Finally, *semantic composition* assesses whether the lexical representation (syntax and semantics) of the complex word can be computed based on the activated lexical representations (syntactic and semantic) of its constituents.

The current study aims to contribute to the existing body of research investigating the distinction between these two final stages, *syntactic licensing* versus *semantic composition* by focusing on the role of aspectual information during lexical access of deverbal formations.

## Deverbal Word Processing

The distinction between *syntactic licensing* and *semantic composition* has been supported by a subsequent body of psycholinguistic (Manouilidou, 2006, 2007; Manouilidou & Stockall, 2014; Tsaprouni & Manouilidou, 2021; Ristić et al., under review) and neurolinguistic (Neophytou et al., 2018; Stockall et al., 2019) research. Specifically, the above psycholinguistic studies were able to tease apart the two processes, i.e. *syntactic licensing* and *semantic composition*, within the same violation paradigm, that is by using pseudowords which violate the selectional restrictions of affixes in terms of either lexical category or argument structure. The same common pattern immerged in a variety of languages, such as English, Greek and Slovenian, providing insights regarding the processing of distinct types of linguistic information, namely lexical category and argument structure, inside deverbal formations. Besides, a more recent study on Bosnian-Croatian-Serbian (BCS) has provided evidence about event structure and semantic well-formedness of such formations (Ristić et al., under review).

Specifically, Manouilidou (2006, 2007) examining Greek suffixes and Manouilidou and Stockall (2014) examining both English prefixes and Greek suffixes have found that argument structure violations (ArgStrViols), such as *kaˈθi-simos* ‘sitt-able’, were accepted more often as grammatical than categorial violations (CatViols), such as *kuverˈti-simos* ‘blanket-able’. The acceptance rates (ARs) were also reflected in reaction times (RTs), given that CatViols were rejected faster than ArgStrViols. This finding clearly points towards the distinction between the two types of constraints with the categorial ones being stronger in comparing to the argument structure ones, which in turn seem to be less sensitive in terms of violability. Thus, lexical category appears to hold a more prominent role than argument structure in the processing of both prefixed and suffixed deverbals. Furthermore, previous studies have also claimed that argument structure constraints apply at a *later stage* of processing compared to categorial constraints. The results were common in all languages investigated and indicate a cross-linguistic way of processing complex pseudowords, either suffixed or prefixed, independently of the individual affixes.

The stimuli of Manouilidou (2007) were also used by Neophytou et al. (2018) in an online visual lexical decision task combined with simultaneous Magnetoencephalography (MEG) data recording. The study confirmed the behavioral evidence in a firmer way. That is, violations of lexical category elicited more activity earlier, in the 200–300 time window, compared to violations of argument structure which elicited activity later, in the 300–500 time window, suggesting that *syntactic licensing* precedes *semantic composition*. Similarly, the effect of CatViol was significant in extended areas of the temporal lobe, whereas the effect of ArgStrViol was mostly detected in the orbitofrontal cortex. Comparable effects were found in Stockall et al. (2019) in a study using prefixes rather than suffixes. Combined results of these two studies provide spatiotemporal grounds for the distinction between the earlier *syntactic licensing* stage, where subcategorization properties are evaluated, and the later *semantic composition* stage, where argument structure information is evaluated.

Of particular interest to the current investigation are two studies conducted in Slovenian, the offline acceptability study of Marjanovič et al. (2013) with young adults, and the study of Manouilidou et al. (2016) with older adults and people with mild cognitive impairment which consists of both offline acceptability judgement and online lexical decision tasks. Both studies, besides lexical category and argument structure, also took into consideration the grammatical aspect of the base verb in deverbal word formations. Therefore, besides CatViols and ArgStrViols, aspectual violations (AspViols) were created. Both studies used the Slovenian suffix –*ec*, which is the rough equivalent to the English suffix –*er*. The suffix –*ec* requires strictly imperfective verbal stems, thus, for the creation of violations, it was combined with perfective verbal stems resulting in pseudowords violating aspectual constraints, namely AspViols, e.g. *preplaˈvalec (from *preplaˈvati* ‘to swim’ [perfective]) compared to the existing word *plaˈvalec* ‘swimmer’ which derives from *ˈplavati* ‘to swim’ [imperfective] (Greenberg, 2006). Results from younger adults (Marjanovič et al., 2013) showed that AspViols, ArgStrViols and CatViols elicited equal ARs. The study by Manouilidou et al. (2016) revealed differences in ARs in older adults and most importantly, higher RTs for AspViols than the rest of the pseudowords, followed by ArgStrViols and then CatViols (Manouilidou et al., 2016). Combined together, these findings suggest that although for Slovenian deverbal pseudowords aspectual rules are as important as categorial and argument structure rules (evidence based on Marjanovič et al., 2013), it seems that it takes longer to process aspectual information compared to information related to argument structure.

To sum up, the existing literature regarding the morphological processing of deverbal formations concludes that in Greek, lexical category and argument structure bear an important role. Particularly, lexical category seems to be processed earlier during *(syntactic) licensing*, whereas argument structure is processed at a later stage of morphological processing during *(semantic) composition*. The role of aspect is not established yet. Thus, the current study aims towards this direction.

### Grammatical Aspect

Since similar studies investigating the same issue have so far only been conducted in Slovenian, a Slavic language, there is a need to clarify the means of expressing aspect in Slovenian as well as in Greek before we proceed to the outline of the current study. To begin with, the main distinction for both language is between perfective and imperfective grammatical aspect. Τhe former is used when the event is accomplished, while the latter is used when the event is ongoing (Mozer, 1994). In Slovenian, imperfective aspect is unmarked, whereas in Greek, it is occasionally unmarked. Perfective aspect in Greek and Slovenian is realized in different ways. More specifically, in Slovenian, the perfective aspect is usually expressed through several distinct prefixes, e.g. ***pre****-ˈplavati* ‘to swim’ [perfective], ***u****-moˈriti* ‘to murder’ [perfective]^1^ (Greenberg, 2006). On the other hand, perfective aspect in Greek is realized mostly through the suffix –*s*– (Hatzidakis, 1905; Hamp, 1961; Koutsoudas, 1962; Babiniotis, 1972; Philippaki-Warburton, 1970, 1973, 1991; Alexiadou & Stavrou, 1998; Ralli, 2003, 2006).

Based on this distinction between perfective and imperfective and going a step further into what is actually considered a perfective stem in derivation, it is necessary to mention that there is a theoretical debate regarding the aspectual properties of the perfective stem in Greek, e.g. *piˈstev-* ‘to believe’ [imperfective]—*piˈsteps-* ‘to believe’ [perfective]. Two recent studies (Revithiadou et al., 2019; Paparounas, 2022) support the idea that the perfective stem is the default one and hence it is aspectually neutral. On the other hand, the vast majority of previous studies we were based on, do not mention underspecificity of stem in terms of aspect (Anastasiadi, 1994; Markantonatou et al., 1996; Anagnostopoulou, 2003; Alexiadou & Anagnostopoulou, 2008; Anagnostopoulou & Samioti, 2013, 2014, Alexiadou, 2018). In contrast, they suggest that the presence of the perfective marker –*s*– in deverbal nominals is linked formally and semantically to perfectivity (Alexiadou & Stavrou, 1998). This well-established view is adopted in the current paper.

The typological differences mentioned above concerning the aspect realization between the two languages, i.e. Greek and Slovenian, raise the question of whether these differences are also present in deverbal word processing.

## The Current Study

The purpose of the current study is to investigate the role of aspect in deverbal word processing in Greek. More specifically, our aim is first to examine whether aspect has an effect in the processing of deverbal formations created with the suffixes *–menos*,* –simos*,* –tos* in Greek, and second, if it does, where aspectual constraints stand in relation to the categorial and the argument structure ones. In general, aspect is an under-investigated type of linguistic information in derivational morphology, and with the exception of the two studies in Slovenian (Marjanovič et al., 2013; Manouilidou et al., 2016), it has never been investigated in any other language in relation to processing of deverbal words.

Following studies of Anastassiadis-Symeonidis (1995); Markantonatou (1995); Markantonatou et al. (1996); Kordoni (2002); Manouilidou (2006); Anagnostopoulou (2003, 2016); Alexiadou and Anagnostopoulou (2008); Anagnostopoulou and Samioti (2013, 2014), Samioti (2015) and Alexiadou (2018) concerning the under examination Greek suffixes, we will describe their individual syntactic and semantic properties. Although they share common features, several differences among them can also be observed. For instance, earlier analyses (Anastassiadis-Symeonidis, 1995; Markantonatou, 1995; Markantonatou et al., 1996; Kordoni, 2002; Manouilidou, 2006) conclude that the suffix –*tos* lacks eventive properties, as it does not completely inherit the properties of the verbal stem, not allowing overt Agent or Instrument complements. Additionally, it is related to non-transparent, idiosyncratic meanings. However, based on the most recent analyses by Anagnostopoulou & Samioti (2014) and Samioti (2015) there are two possible interpretations of –*tos*, one denoting “ability/possibility”, in examples such as *fouskoˈtos* ‘inflatable’ and one denoting “characteristic state”, such as in *vraˈstos *‘boiled’. These two interpretations entail different event involvements and distinct event participants. For instance, in the “ability/possibility” reading, a potential Agent and a Theme arguments are implied, while in the “characteristic state” reading no Agent, but just a Theme argument is assumed.^2^ Nonetheless, if one notices the type of verbs that are compatible with the “ability/possibility” reading, we see that they are mostly psychological or perception verbs (e.g. *piste΄ftos* ‘believable’, *agapi΄tos* ‘beloved’, *ipofer΄tos* ‘tolerable etc.). These verbs are not compatible with a prototypical Αgent, but rather with an Experiencer. On the other hand, the suffix –*menos* is traditionally considered as having more event properties compared to –*tos*. However, it is also associated with two readings reflecting two possible syntactic analyses (Anagnostopoulou, 2003; Anagnostopoulou & Samioti, 2014)^3^ which further means that it does not always have clear event implications. Specifically, –*menos* can create adjectives that denote a “result state”, e.g. *skotoˈmenos* ‘killed’ and it can also create adjectives that denote a “target state” which are in principle reversible, e.g. *kriˈmenos* ‘hidden’. The “result state” involves both an Agent and a Theme argument, as they license agentive modifications. This is not the case for the “target state”, which only involves the Theme argument. Finally, the suffix –*simos* does not show the polysemy the other two suffixes do, and it is not open to various readings. The suffix –*simos* denotes “possibility/ability” and it always involves an Agent and a Theme event participants.

The suffix –*menos*, discussed in this paper, is different from the suffix –*omenos*, which is always stressed at the antepenultimate syllable and attaches strictly to imperfective verbal stems bearing the meaning of an action that happens continuously. The suffix –*menos*, on the other hand, is always stressed at the penultimate syllable and bears a perfective reading describing an action that has been completed (Holton et al., 1997; Kleris & Babiniotis, 2005). Moreover, the suffix –*omenos* is compatible with unergative verbs, e.g. *ˈtrexo* ‘to run’ < *treˈxumenos* ‘running’, whereas the –*menos* is compatible with verbs of the following argument structure < Agent < Theme>> (Manouilidou, 2006).

The above theoretical studies have subsequently provided insights regarding the derivational restrictions of each suffix. Based on these, we created the stimuli of the present study, as our intention was to further explore whether these distinct features affect the processing of grammatical aspect. To this end, the violation paradigm was used in two experiments, an offline acceptability judgement task and an online lexical decision task.

The purpose of the offline grammaticality rating task was to test participants’ knowledge of word formation rules regarding deverbal formations formed with the suffixes –*menos*,* –simos*, and *–tos*, with a focus on the status of aspectual rules in speakers’ perception of deverbal formations. On the other hand, the purpose of the online lexical decision task was to investigate the automatic morphological processing of deverbal formations that takes place upon word viewing.

In previous studies (Manouilidou, 2006, 2007; Marjanovič et al., 2013; Manouilidou & Stockall, 2014; Manouilidou et al., 2016), the differences between conditions at the level of ARs were interpreted as reflecting the different status of the distinct derivational rules in speakers’ perception. Particularly, low ARs were interpreted as evidence of a strong type of derivational constraints, whereas high ARs were considered to indicate a less strong type of derivational constraints. On the other hand, differences between conditions at the level of RTs were interpreted as reflecting the level of difficulty for the speakers to process these types of violations and consequently the specific types of linguistic information that are taken into account during deverbal word processing. More specifically, low RTs were taken as a sign of an easier and thus faster processing, possibly reflecting *licensing*, whereas high RTs were thought to reveal a more difficult and thus slower processing, possibly pointing towards *semantic composition*. The current data will be interpreted within this frame.

Based on previous behavioral studies, we would expect a common pattern across suffixes, unless their individual properties prove to be strong enough to elicit distinct patterns of processing when it comes to aspect. Concerning ARs, we expect the following continuum: CatViols < AspViols < ArgStrViols < GramWs, whereas concerning RTs, we expect the following: GramWs < CatViols < AspViols < ArgStrViols. Considering that the perfective aspect is a common feature among all deverbal formations created by the three under examination suffixes, just like the verbal stem of the lexical category, we proceed to the following hypothesis: the parser initially recognizes the lexical category of the derivational stem, then checks the type of aspect and afterwards evaluates whether the argument structure properties of the verbal stem match with the suffix’s individual derivational restrictions. Thus, we expect that AspViols both in terms of ARs and RTs will be after CatViols and before ArgStrViols.

## Experiment 1: Offline Acceptability Judgement Task

### Method

#### Participants

A total of 207 individuals participated in the experiment, all of them were adults, native speakers of Greek. Their mean age was 32,6 years old, whereas 34,3% of them were male and 65,7% of them were female. Regarding their level of education, 4,8% of them had a PhD, 40,09% of them had a Master’s degree, 38,16% of them had a Bachelor’s degree, 2,89% of them had graduated from a technical school and 14,06% of them had graduated from high school.

### Materials

The experimental stimuli consisted of a total of 558 pseudowords: 60 Aspectual Violations (AspViols) (20 per suffix), 131 Categorial Violations (CatViols), 188 Argument Structure Violations (ArgStrViols) and 179 Novel Words (NovWs) formed with the following three suffixes: *–menos*, *–simos*, *–tos.* Previous relevant studies (Manouilidou, 2006, 2007; Manouilidou & Stockall, 2014; Neophytou et al., 2018), reported discrepancies at the level of both ARs and RTs between CatViols and ArgStrViols created with these suffixes. Examples of each type of pseudoword are provided in Table 1.

### Categorial Violations (CatViols)

Assuming that stems are stored with a categorial feature (Aitchison, 1994; Sandra & Taft, 2014), they are divided into nominal, e.g. *kaˈrekl-a* ‘chair’, adjectival, e.g. *ˈomorf-os* ‘beautiful’, and verbal ones, e.g. *aˈniγ-o* ‘to open’. Given that all suffixes we used select a verbal stem, CatViols were created by adding the specific suffixes to either nominal or adjectival stems instead of verbal.

### Aspectual Violations (AspViols)

AspViols were created by adding the specific suffixes to the opposite aspect of what they typically attach to during the derivation process. That is, while all three suffixes, despite a few exceptions, normally attach to perfective verbal stems, for the violations, we had them paired with imperfective stems. One issue that came up when forming these pseudowords, was that in most cases, this combination of imperfective stem + suffix resulted in successive consonants that were hard to pronounce. In this case, we had two choices; either to add the linking vowel -o- to make them more pronounceable, e.g. ˈ*ðin-o ‘give’ > ˈ*ð*in-o-ˈmenos ‘given’* or to opt for the assimilated form *ðiˈmenos (*ðinˈmenos > * ðiˈmenos - ˈðin- resulting in *ˈði-* in front of –*menos*). Formations with total assimilation would probably sound more euphonic. On the other hand, assimilation would make stem identification less transparent and thus, harder for the participants to identify it, since it would be in a reduced form. Given that the study is based on the premise that participants recognize the constituent morphemes of pseudowords and then check whether they can fit with each other, not having transparent stems would undermine the purpose of the study.

### Argument Structure Violations (ArgStrViols)

In general, the suffixes –*menos*, –*simos* and –*tos* require verbal stems with Agent external and Theme internal argument structure (< Agent < Theme>> ) (Manouilidou, 2006). Thus, ArgStrViols were primarily created by adding these suffixes to unergative stems, the argument structure of which requires an Agent external argument (< Agent> ). In addition, ArgStrViols were also created by combining the suffixes under investigation with verbal stems that are not compatible with their individual restrictions of attachment (Kordoni, 2002; Anagnostopoulou, 2003, 2016; Alexiadou & Anagnostopoulou, (2008; Anagnostopoulou & Samioti, 2013, 2014; Samioti, 2015; Alexiadou, 2018) (see Section “The current study”). For instance, the suffix –*simos* is not compatible with verbs that cannot combine with middle voice (Alexiadou, 2018). The suffix –*menos* cannot be attached to verbs denoting a standard situation (Anagnostopoulou, 2003), and the suffix –*tos*, when meaning ability, is not compatible with verbalizers (Markantonatou et al., 1996; Manouilidou, 2006).

### Novel Words (NovWs)

NovWs were created by combining the suffixes with verbal stems that are compatible with their individual restrictions of attachment, but they are nonetheless unattested.

**Table 1:** Material examples across experimental conditions and suffixes

Stem	Suffix	
Categorial Violations (CatViols)		
ˈ ð*edro* ‘tree’ +	–*menos* =	*ðedroˈmenos ‘tree-ed’
ˈð*iskolos* ‘difficult’ +	–*simos* =	*ðiskoˈlosimos ‘difficult-able’
*kaˈrekla* ‘chair’ +	–*tos* =	*kareklaˈtos ‘chair-able’
Aspectual Violations (AspViols)		
*aroˈsteno* ‘to be sick’ [imperfective] +	*–menos* =	*arosteˈmenos ‘sicked’
*eˈkleγο* ‘to elect’ [imperfective] +	–*simos* =	*ekleˈγosimos ‘elect-able’
*aˈniγο* ‘to open’ [imperfective] +	–*tos* =	*aniγoˈtos ‘open-able’
Argument Structure Violations (ArgStrViols)		
*ˈepiviˈono* ‘to survive’ +	*–menos* =	*epivioˈmenos ‘survived’
*koliˈbo* ‘to swim’ +	*–simos* =	*koliˈbisimos ‘swim-able’
*ˈtrexo* ‘to run’ +	*–tos* =	*treˈxtos ‘runed’
Novel Words (NovWs)		
*kiˈto* ‘to look’ +	*–menos* =	*kitaˈmenos ‘looked’
*çereˈto* ‘to greet’ +	*–simos* =	*çereˈtisimos ‘greet-able’
*sinaˈdo* ‘to meet’ +	*–tos* =	*sinadiˈtos ‘meet-able’

### Procedure

Participants were asked to decide whether or not the presented words exist in their language, i.e., Greek, by choosing YES or NO. Stimuli were divided into three (3) lists of 186 items each and they were randomized in each experimental trial. Each participant saw one list, that is, each list was completed by 69 participants.

### Data Analysis

Statistical analysis was performed in R Studio using the *glmer* function of the lme4 package in R for coefficients’ estimation (Bates & Maechler, 2009). The dependent variable of Response was coded for each trial. Each response was coded for whether participants answered positively with a YES (1) or negatively with a NO (0), as well as whether they answered correctly (1) or incorrectly (0) to the question whether or not the presented string is an existing word. Finally, we checked whether there were responses deviating more than 2.5 of the standard deviations from the mean response for each participant x condition in order to be excluded from analysis. However, none of the responses were deviating more than 2.5 of the standard deviations of the mean response, so there was no removal of this type.

The initial model (off.acc.m.1), based on Barr et al. (2013), had Acceptance Rates (ARs) as the dependent variable, and Condition (AspViol, ArgStrViol, CatViol, NovW, GramW) x Suffix *(–menos*,* –simos*,* –tos*) as fixed factors. As random effects were the maximum possible structure, which included a random intercept for item, a random intercept for subject, and a random intercept and slope corresponding to the fixed effects structure for both item and subject including all correlations.

- (off.acc.m.1) glmer (response ~ condition * suffix + (1 + condition * suffix | item) + (1 + condition * suffix | subject)).

By-subject and by-item random slopes and intercepts were included by default. However, this model failed to converge with this structure, so the random effects’ structure was simplified until the model converged. Thus, as fixed effects of the final generalized linear mixed model (off.acc.m.2) (Baayen et al., 2008) with family = binomial were taken Condition, Suffix, as well as the interaction between the two. Both the variables were coded with the default treatment contrasts, with –*menos* as the baseline for the Suffix variable and AspViol as the baseline for the Condition variable. As random effects of the model were considered by-Subject and by-Item intercepts.

- (off.acc.m.2) glmer (response ~ condition * suffix + (1 | item) + (1 | subject)).

Finally, pairwise comparisons were performed using the *emmeans* package in R, with Tukey method for multiple comparison corrections. In order to perform comparisons of the AspViol condition with the rest of the experimental material (ArgStrViol, CatViol, NovW, GramW), as well as comparisons of the distinct types of suffixes *(–menos*,* –simos*,* –tos*) within the condition of AspViol, we used the following formulas: *pairs (emmeans (model()*,* “Condition”*,* by = “Suffix”))*,* pairs (emmeans (model()*,* “Suffix”*,* by = “Condition”)).*

## Results

ARs indicate how many times participants decided that the presented word was a real Greek word, and they were calculated for all three violation conditions for each of the three suffixes separately (Table 2).

**Table 2:** Percentages of ARs per condition, within each suffix and across suffixes

Condition	–*menos* (%)	–*simos* (%)	–*tos* (%)	Overall (%)
CatViols	10	9	8	9
ArgStrViols	29	34	18	28
AspViols	14	10	4	9
NovWs	44	42	29	37

The summary of the best fitting model (off.acc.m.2) is in Table 3.

**Table 3:** Summary of the best fitting generalized linear mixed model for ARs (off.acc.m.2) (AspViol served as baseline for the comparisons among conditions, and AspViols of *–menos* served as baseline for comparisons among suffixes within the AspViol condition)

	Estimate	Std. Error	z value	Pr(>|z|)	
(Intercept)	−2.1648	0.1254	−17.267	< 2e–16	***
Condition (CatViols)	−0.1232	0.1829	−0.674	0.987054	
Condition (NovWs)	2.0168	0.1721	11.721	1.39e–10	*** (a)
Condition (ArgStrViols)	1.2692	0.1685	7.531	2.12e–05	*** (a)
Suffix ( *–* *simos* )	−0.4152	0.1250	−3.321	0.551598	
Suffix ( *–* *tos* )	−1.5000	0.1633	−9.188	0.000165	*** (b)

Significant effects are marked with an asterisk (*)

Effects that are statistically significant are noted with a *, and are as follows: (a) AspViols differ from NovWs and ArgStrViols evoking lower ARs but there is no difference compared to CatViol (b) a difference between –*menos* and –*tos*, such that –*menos* AspViols had higher ARs than *–tos* AspViols, and no difference between -*menos* and -*simos* AspViols.

Pairwise comparisons of condition by suffix, as well as of AspViol by suffix were carried out and are presented in Tables 4 and 5.

**Table 4:** Pairwise comparisons of conditions by Suffix

Suffix	Contrast	Estimate	SE	df	z.ratio	*p*.value
– *menos*	AspViols – CatViols	0.00597	0.368	Inf	0.016	1.0000
	AspViols – NovWs	−2.40247	0.374	Inf	−6.417	< 0.0001
	AspViols – ArgStrViols	−1.48398	0.349	Inf	−4.252	0.0001
– *simos*	AspViols – CatViols	−0.01289	0.373	Inf	−0.035	1.0000
	AspViols – NovWs	−2.51935	0.341	Inf	−7.380	< 0.0001
	AspViols – ArgStrViols	−2.02563	0.337	Inf	−6.017	< 0.0001
– *tos*	AspViols – CatViols	−1.07692	0.372	Inf	−2.895	< 0.0198
	AspViols – NovWs	−3.09343	0.350	Inf	−8.834	< 0.0001
	AspViols – ArgStrViols	−2.16379	0.367	Inf	−5.894	< 0.0001

Pairwise comparisons, brought into light a significant difference between AspViols and CatViols for the suffix *–tos*, which makes it the only suffix for which AspViols differ from CatViol.

**Table 5:** Pairwise comparisons of suffixes for AspViol

Condition	Contrast	estimate	SE	Df	z.ratio	*p*.value
AspViols	– *menos* / – *simos*	0.233	0.391	Inf	0.595	0.8226
	– *menos* / – *tos*	1.533	0.407	Inf	3.767	0.0005
	– simos / – *tos*	1.301	0.412	Inf	3.155	0.0046

Pairwise comparisons show that within AspViols the suffix *–tos* differs from both *–menos* and *–simos*, whose ARs appear to be equal.

Furthermore, pairwise comparisons between AspViols formed with the additional vowel *–*o*–* and those that do not (see Section “Materials” – AspViols), showed no statistically significant difference (*β* = 0.00378, *SE* = 0.345, *df* = Inf, *z.ratio* = 0.032, *p. value* = 1.0000).

### Discussion: Offline Acceptability Judgement Task

Results of Experiment 1 revealed that the role of aspectual rules differs depending on the specific suffix. Specifically, AspViols formed with *–tos* significantly differed from all types of non-attested pseudowords (CatViol, ArgStrViol, NovWs), being more robustly rejected. In addition, ARs of AspViols formed with *–tos* were lower to those of *–menos* and *–simos.* The suffix *–tos* is the only suffix among the three whose AspViols have lower ARs than its CatViols.

Based on the rationale of the study design (see Section “The current study”), we could conclude that aspectual constraints are stronger than the categorial ones in *–tos* formations. However, the assumption that aspect holds a more prominent role than lexical category in any sort of deverbal formation would be groundbreaking and somehow surprising. Thus, considering that that *–tos* has weak eventive properties (see Section “The current study”), we could assume that other factors might come into play and hence more research is needed to understand the underlying reasons behind that finding. Probably the RTs results of Experiment 2 will shed more light to this surprising finding.

On the other hand, in pseudowords formed with *–menos* and *–simos* while AspViols differ significantly from ArgStrViols and NovWs, there is no difference between AspViols and CatViols. There is a continuum in the ARs, but for these two suffixes, the effect of AspViols cannot be distinguished from the effect of CatViols (as it is the case in pseudowords formed with *–tos*) with both being judged as equally robust.

This finding is not entirely in line with Marjanovič et al. (2013) which reports on data from Slovenian selected from a comparable population to ours. Namely, in Slovenian deverbal formations, aspectual rules were judged of equal importance to the rules imposed by both lexical category and argument structure. In Greek, unlike Slovenian, aspectual rules clearly differ from those of argument structure concerning their violability. whereas depending on the suffix *(–tos* vs. *–simos & –menos*) they can also differ from CatViols as well.

Thus, it is clear that in Greek, unlike Slovenian, aspectual rules bear a stronger status than argument structure rules in deverbal word formations, which means that the presence of a verbal stem of a perfective nature constitutes a prerequisite for deverbal formations to be built and to be considered grammatical.

### Experiment 2: Online Lexical Decision Task

#### Participants

A total of 76 participants, 36 male and 40 female, participated in the experiment. Each of the three lists was seen by approximately 25 participants. All of them were adults, native speakers of Greek. Their mean age was 33,05 years. 47% of them were male and 53% of them female. Regarding their level of education, 2,3% of them had a PhD, 33,5% had a Master’s degree, 40,2% had a Bachelor’s degree, and 24% of them had high school education.

### Materials

The experimental stimuli consisted of 240 pseudowords and 225 existing words (GramWs). For GramWs, we used the Anastasiadi-Simeonidi’s *Reversed Dictionary of the Modern Greek Language* (https://www.greek-language.gr/greekLang/modern_greek/tools/lexica/reverse/index.html). Pseudowords consisted of 60 AspViols (20 per suffix), 60 CatViols (20 per suffix), 60 ArgStrViols (20 per suffix) and 60 NovWs (20 per suffix). Stimuli were divided into three lists of 75 GramWs and 80 pseudowords each, and they were randomized in each experimental trial.

For AspViols, the same dataset as in Exp. 1 was used, whereas in the rest of the experimental conditions, stimuli were chosen by following a matching procedure. Specifically, items were matched across conditions based on the average ARs of Exp. 1, by excluding the lowest and highest ARs. Given the differences observed in Exp. 1 between suffixes, we made an effort to match stem frequencies among suffixes and not necessarily among conditions. This is something we took into account in the analysis (see section ‘Data Analysis’ below). Despite this effort, in AspViols, stem frequency of *–tos* was slightly higher compared to the stem frequency of *–simos* and *–menos*. For frequency measures the *ILSP Psycholinguistic Resource (*http://speech.ilsp.gr/iplr/NumTool.aspx*)* was used. Material properties are illustrated in Table 6.

**Table 6:** Stem frequencies per condition and suffix

Condition	Suffix	Stem frequency	ARs of Exp.1 (%)
CatViols	*simos*	16.85	9
	–*menos*	16.97	10
	–*tos*	16.64	9
ArgStrViols	–*simos*	11.07	34
	–*menos*	11.04	30
	–*tos*	10.00	18
AspViols	–*simos*	0.10	10
	–*menos*	0.14	14
	–*tos*	3.13	4
NovWs	–*simos*	1.85	42
	–*menos*	1.48	44
	–*tos*	1.87	31

### Procedure

The stimulus presentation was designed in OpenSesame and was written in a Javascript code (ECMA 5.1.). The font was Times New Roman, size 24 in black colour and the background was white. Before each stimulus presentation, a fixation cross was presented for 500 ms. Participants were asked to decide as fast and as accurately as possible whether or not the presented words exist in their language, i.e., Greek, by responding YES or NO.

### Data Analysis

Statistical analysis was performed in R Studio using the *glmer* and the *lmer* functions of the lme4 package in R for coefficients’ estimation (Bates & Maechler, 2009). Responses with a RT below 200ms and above 4000ms were removed, resulting in a total removal of 7.5% of the data. For the RT analysis, incorrect trials were removed resulting in a total removal of 15.5% of the data.

Finally, we checked whether there were RTs deviating more than 2.5 of the standard deviations from the mean reaction time for each participant by condition in order to be excluded from the analysis. The same procedure was adopted for the YES/NO responses. However, neither responses nor reaction times were deviating more than 2.5 of the standard deviations of the mean response/reaction time, so there was no further removal.

The initial models (onl.acc.m.1; rt.m.1), based on Barr et al. (2013), had ARs and RTs as dependent variables, and Condition (AspViol, ArgStrViol, CatViol, NovW, GramW) by Suffix *(–menos*,* –simos*,* –tos*), the interaction between the two, stem frequency, as well as number of item’s phonological neighbours (*N_phon_neigh_item*) for ARs analysis and item’s length for RTs analysis as fixed factors, respectively. Initially, all possible psycholinguistic variables^4^ were included into the model, however, only the variables of *N_phon_neigh_item* for ARs and *item’s length* for RTs proved to be significant. As random effects were the maximum possible structure, which included a random intercept for item, a random intercept for subject, and a random intercept and slope corresponding to the fixed effects structure for both item and subject including all correlations. By-subject and by-item random slopes and intercepts were included by default.

- (onl.acc.m.1) glmer (response ~ condition * suffix + N_phon_neigh_item + stem frequency + (1 + condition * suffix | item) + (1 + condition * suffix | subject) + (1 + N_phon_neigh_item | item) + (1 + N_phon_neigh_item | subject).
- (rt.m.1) lmer (RT ~ condition * suffix + item’s length + stem frequency + (1 + condition * suffix | item) + (1 + condition * suffix | subject) + (1 + item’s length | item) + (1 + item’s length | subject).

However, these models failed to converge with this structure, so different optimizers were used separately via the *allFit* function. The models failed to converge even after the addition of the optimizers. Therefore, the random effects’ structure was simplified until the models converged. The final simplified models (onl.acc.m.2; rt.m.2) had as fixed effects Condition, Suffix, the interaction between them, number of phonological neighbours for ARs analysis and item’s length for RTs analysis, whereas random effects were by-Subject and by-Item intercepts.

- (onl.acc.m.2) glmer (response ~ condition * suffix + N_phon_neigh_item + (1 | item) + (1 | subject)).
- (rt.m.2) lmer (RT ~ (condition * suffix) + item’s length + (1 | item) + (1 | subject)).

Finally, pairwise comparisons were performed using the *emmeans* package in R, with Tukey method for multiple comparison corrections. In order to perform simple comparisons of the AspViol condition with the rest of the experimental material (ArgStrViol, CatViol, NovW, GramW), as well as comparisons of the distinct suffixes *(–menos*,* –simos*,* –tos*) within the condition of AspViol, we used the following formulas: *pairs (emmeans (model()*,* “Condition”*,* by = “Suffix”))*,* pairs (emmeans (model()*,* “Suffix”*,* by = “Condition”)).*

## Results

### Acceptance Rates (ARs)

Percentages of ARs are presented in Table 7.

**Table 7:** Percentages of ARs per condition, within each suffix and across suffixes

Condition	–*menos* (%)	–*simos* (%)	–*tos* (%)	Overall (%)
CatViols	7	7	9	8
ArgStrViols	24	22	16	21
AspViols	14	11	10	12
NovWs	36	30	30	32
GramWs	92	85	85	87

The summary of the best fitting model (onl.acc.m.2) is in Table 8.

**Table 8:** Summary of the best fitting generalized linear mixed model (onl.acc.m.2) for ARs (AspViol served as baseline for the comparisons among conditions, and AspViols of *–menos* served as baseline for comparisons among suffixes within the AspViol condition). Significant effects are marked with an asterisk (*)

	Estimate	Std. Error	z. ratio	Pr(>|z|)	
(Intercept)	−2.25655	0.28743	−7.851	4.13e–15	***
Condition (CatViols)	−0.72295	0.39425	−1.834	0.0667	.
Condition (GramWs)	4.13743	0.33193	12.465	< 2e-16	*** (a)
Condition (NovWs)	1.51603	0.35825	4.232	2.32e–05	*** (a)
Condition (ArgStrViols)	0.73017	0.36445	2.203	0.0451	* (a)
Suffix ( *–* *simos* )	−0.13676	0.37875	−0.361	0.7180	
Suffix ( *–* *tos* )	−0.24204	0.37719	−0.642	0.5211	
N_phon_neigh_item	0.44247	0.05171	8.556	< 2e–16	*** (b)

The main findings can be summarized as follows: (a) AspViols differ from all conditions except from CatViol evoking lower ARs than GramWs, NovWs, and ArgStrViols, (b) an effect of item’s number of phonological neighbours with a positive correlation (0.48), meaning that the greater the number of item’s phonological neighbours, the greater the ARs. No other psycholinguistic variable had significant effects.

Additionally, a comparison between ArgStrViols vs. CatViols also yielded a significant difference (*β* = 1.356, *SE* = 0.184, *df* = Inf, *z. ratio =* 7.358, *p. value = <* 0.0001), suggesting that even CatViols might be at the end of the continuum of acceptance, followed by AspViols and then ArgStrViols.

Pairwise comparisons of conditions by suffix were carried out as well and they are presented in Table 9.

**Table 9:** Pairwise comparisons of conditions by suffix for ARs

Suffix	Contrast	Estimate	SE	Df	z.ratio	*p*.value
– *menos*	AspViols – CatViols	0.723	0.394	Inf	1.834	0.3540
	AspViols – GramWs	−4.137	0.332	Inf	−12.465	< 0.0001
	AspViols – NovWs	−1.516	0.358	Inf	−4.232	0.0002
	AspViols – ArgStrViols	−0.730	0.364	Inf	−2.003	0.2642
	ArgStrViols – CatViols	1.4660	0.377	Inf	3.884	0.0100
– *simos*	AspViol – CatViols	0.644	0.396	Inf	1.628	0.4790
	AspViol – GramWs	−3.692	0.304	Inf	−13.013	< 0.0001
	AspViol – NovWs	−1.321	0.358	Inf	−3.688	0.0021
	AspViol – ArgStrViols	−0.902	0.361	Inf	−2.499	0.0907
	ArgStrViol – CatViols	1.522	0.369	Inf	4.127	0.0040
– *tos*	AspViol – CatViols	0.251	0.384	Inf	0.653	0.9661
	AspViol – GramWs	−3.955	0.313	Inf	−12.634	< 0.0001
	AspViol – NovWs	−1.403	0.352	Inf	−3.981	0.0007
	AspViol – ArgStrViols	−0.535	0.365	Inf	−1.468	0.5836
	ArgStrViol – CatViols	0.772	0.364	Inf	2.1200	0.2114

Pairwise comparisons of condition by suffix show that, across all suffixes, AspViols do not differ neither from CatViols nor from ArgStrViols, as the p-values computed are higher than the alpha significance level. However, ArgStrViols differ from CatViols for *–simos* and *–menos* but not for *–tos.*

### Reaction Times (RTs)

RTs were calculated for all three violation conditions for each of the three suffixes separately, as well as across suffixes (see Table 10).

**Table 10:** Mean RTs (ms) and SDs per condition, within each suffix and across suffixes

Condition	–*menos*	–*simos*	–*tos*	Overall
CatViols	1474 ms (689)	1450 ms (696)	1334 ms (688)	1419 ms (693)
ArgStrViols	1749 ms (815)	1536 ms (758)	1423 ms (719)	1563 ms (774)
AspViols	1526 ms (746)	1735 ms (762)	1314 ms (667)	1516 ms (744)
NovWs	1750 ms (788)	1616 ms (820)	1574 ms (760)	1641 ms (792)
GramWs	1310 ms (657)	1412 ms (702)	1270 ms (662)	1329 ms (676)

The summary of the best fitting model (rt.m.2) is in Table 11.

**Table 11:** Summary of the best fitting linear mixed model (rt.m.2) for RTs (AspViol served as baseline for the comparisons among conditions, and AspViol *–menos* served as baseline for comparisons among suffixes within the AspViol condition)

	Estimate	Std. Error	Df	t value	Pr(>|t|)	
(Intercept)	975.720	87.140	396.34	11.197	< 2e-16	***
Condition (CatViols)	−156.628	65.408	410.48	−2.395	0.017084	* (a)
Condition (GramWs)	−353.627	53.191	428.25	−6.648	9.06e-11	*** (a)
Condition (NovWs)	109.792	70.367	509.98	1.560	0.119313	
Condition (ArgStrViols)	134.159	67.646	453.95	1.983	0.047941	* (a)
Suffix ( *–* *simos* )	9.304	67.756	429.33	0.137	0.890844	
Suffix ( *–* *tos* )	−179.44	65.657	406.14	−2.733	0.006549	** (b)
Item’s length	64.557	6.078	405.27	10.622	< 2e-16	*** (c)

Significant effects are marked with an asterisk (*)

The main findings can be summarized as follows: (a) an effect of Condition, such that AspViols evoking longer RTs than CatViols, and marginally shorter RTs than ArgStrViols, (b) a significant longer RTs for AspViols formed with the suffix –*menos* compared to AspViols formed with the suffix –*tos* but no difference between *–menos* and –*simos* (c) an effect of item’s length with a negligible correlation (0.14).

**Table 12:** Pairwise comparisons of conditions by Suffix for RTs

Suffix	Contrast	estimate	SE	Df	z.ratio	*p*.value
– *menos*	AspViols – CatViols	156.6	65.4	Inf	2.395	0.1167
	AspViols – GramWs	353.6	53.2	Inf	6.648	< 0.0001
	AspViols – NovWs	−109.8	70.4	Inf	−1.560	0.5231
	AspViols – ArgStrViols	−134.2	67.6	Inf	−1.983	0.2741
	CatViols – ArgStrViols	−290.8	65.8	Inf	−4.418	0.0001
– *simos*	AspViols – CatViols	223.8	64.8	Inf	3.452	0.0050
	AspViols – GramWs	301.9	52.3	Inf	5.771	< 0.0001
	AspViols – NovWs	−19.4	69.2	Inf	−0.280	0.9987
	AspViols – ArgStrViols	52.0	67.6	Inf	0.770	0.9393
	CatViols – ArgStrViols	−171.8	65.3	Inf	−2.630	0.0650
– *tos*	AspViols – CatViols	33.0	63.5	Inf	0.519	0.9855
	AspViols – GramWs	105.2	51.2	Inf	2.056	0.2394
	AspViols – NovWs	−255.8	65.8	Inf	−3.887	0.0010
	AspViols – ArgStrViol	−151.5	63.9	Inf	−2.371	0.1233
	CatViols – ArgStrViols	−184.5	64.0	Inf	−2.884	0.0321

The effects outlined in Table 11 do not survive in pairwise comparisons among conditions within each suffix separately (Table 12). Particularly, no difference occurred for the pairs of AspViols – ArgStrViols across all suffixes, as well as AspViols – CatViols for the suffixes –*menos* and –*tos*. On the other hand, a comparison between ArgStrViols vs. CatViols yielded a significant difference for the suffixes –*menos* and –*tos*, suggesting once again that CatViols might be at the fastest end of the continuum of processing speed, followed by AspViols and then ArgStrViols, a pattern that matches ARs.

**Table 13:** Pairwise comparisons of suffixes by condition for RTs

Condition	Contrast	estimate	SE	Df	z.ratio	*p*.value
AspViols	*–* *menos /* – *simos*	−9.30	67.8	Inf	−0.137	0.9897
	– *menos /* – *tos*	179.44	65.7	Inf	2.733	0.0173
	– *simos /* – *tos*	188.75	68.8	Inf	2.742	0.0168

Pairwise comparisons within AspViols (Table 13) reveal that AspViols with the suffix –*tos* yielded significantly faster RTs compared to AspViols formed with –*simos* and –*menos.*

#### Discussion: Online Lexical Decision task

After obtaining patterns of acceptability in Experiment 1, Experiment 2 targeted the online processing of deverbal formations in order to determine accuracy and RTs patterns in a chronometrized task. The overall results of Experiment 2 at the level of ARs suggest that AspViols do not differ from either CatViols or ArgStrViols. This finding is not completely in line with the results of Experiment 1, in which we saw that AspViols differed from ArgStrViols for all three suffixes and that they also differed from CatViols for *–tos.* This suggests a task effect, and thus implying that under time pressure AspViols appear to stand equally for all suffixes.

When considering all pseudowords, CatViols differ significantly from ArgStrViols (with the exception of *–tos*), suggesting that there might be a continuum in acceptance, ranging from CatViols, to AspViols and then to ArgStrViols for **–***simos* and **–***menos*. The distinction between CatViols and ArgStrViols is in accordance with previous studies (Manouilidou, 2006, 2007; Manouilidou & Stockall, 2014; Neophytou et al., 2018), which clearly dissociate CatViols from ArgStrViols. The current results also incorporate AspViols in this continuum, suggesting that it is between the two other types of violations. This effect, though, as mentioned, is not consistent across suffixes. Namely, while the difference between CatViols and ArgStrViol remain for *–simos* and *–menos*, we see no difference on ARs between AspViols and the other two types of violations (see Table 9).

When it comes to RTs, we identify two different patterns. Within the suffixes **–***menos* and **–***tos* AspViols do not differ from either CatViols or ArgStrViols, whereas CatViols seem to be processed faster than ArgStrViols. Therefore, it seems that the processing of aspect could be situated in a time window between the processing of lexical category and argument structure. On the other hand, within the suffix **–***simos* AspViols do not differ from ArgStrViols, neither do ArgStrViols differ from CatViols, whereas CatViols are significantly faster than AspViols (see Table 12).

Taking the above findings into account, we can conclude that the processing of deverbal formations is carried out in a different way across suffixes. In formations created with the suffixes –*menos* and –*tos* the processing of aspectual information remains unclear, as we cannot place it to a specific time frame. On the other hand, in formations created with the suffix –*simos* aspect and argument structure are possibly processed at the same time frame (at the stage of *semantic composition*) after the processing of lexical category has taken place. If we assume that for –*simos* the processing of lexical category takes place at the stage of *syntactic licensing*, then, we have grounds to believe that the processing of *aspect* takes place at the stage of *semantic composition*, same as argument structure processing. Of course, this leaves an open question as to why the same pattern was not observed for the other two suffixes, i.e. –*menos* and –*tos*. What might be relevant is the existence of two possible readings for –*tos* and –*menos* formations and the possibility of having only one event participant (Theme).

One way to address the difference among suffixes is to consider the fact that AspViols of the suffix –*tos* were significantly faster compared to the ones with the suffixes –*menos* and –*simos.* This fact might indicate that in adjectives formed with the suffix –*tos*, namely those that lack event implication (see Section “The current study”), aspect is processed faster or not at all compared to adjectives formed with –*menos* and –*simos.* This difference could also be attributed to other factors as well, such as the high frequency of the stems used for –*tos* AspViols (see Table 6). AspViols of the suffix –*tos* are easier to process and thus they are rejected faster compared to the rest of experimental conditions formed by the same suffix. Finally, as mentioned also earlier (see Section “Discussion – Offline Acceptability Judgement Task”), we came across the surprising finding that aspectual rules hold a stronger status within the suffix *–tos* compared to the rest of the suffixes, making them easier to reject. Thus, the distinction of *–tos* from *–menos* and *–simos* regarding the processing of aspect is reflected both at the level of RTs and the offline ARs.

Taking together the results of both ARs and RTs, we could conclude that the role of the specific suffix is stronger than originally anticipated. Namely, for an unambiguous deverbal suffix such as –*simos*, which has event implications and always includes an Agent and a Theme argument, aspect is processed later than lexical category despite being equally strong to speakers’ perception (as indicated by ARs). For polysemous deverbal suffixes such as –*tos* and –*menos,* which do not always entail clear event implications, aspect does not differ from the other types of information that need to be processed during lexical access.

#### General Discussion

Aspect is an important feature in deverbal word formations, imposing significant constraints. With the present study, we wanted to investigate its role in lexical access of deverbal word formations and bring into light its processing correlates. Similarly, and from a processing perspective, we also wanted to further inform theories and models of lexical access, especially the ones that postulate stages during this process by asking the question whether and when in time information regarding aspect is accessed. With respect to “whether” we process aspectual information, we considered data from an acceptability judgment task (Experiment 1) as well as accuracy rates from a lexical decision task (Experiment 2). With respect to “when”, we considered data from the lexical decision task (Experiment 2). We will address these two questions separately and we will comment on two important issues that is, a task effect and individual differences among suffixes. The main finding with respect to the first question, is that there is an important task effect, given that the results of the two experiments do not agree with each other. That is, while the offline acceptability judgment task showed that speakers treat AspViols as being different from violations of both CatViols and ArgStrViols (for the suffix –*tos*) and different from ArgStrViols (for the suffixes –*menos* and –*simos*), this difference did not surface in the online lexical decision task, in which no difference between AspViols and other types of violations was observed in terms of accuracy, suggesting that aspectual constraints cannot be clearly dissociated either from the ones of lexical category nor from argument structure.

When it comes to the second question concerning RTs, the main finding of the whole study is that there are important individual differences among suffixes that should be taken into account. For instance, the suffix **–***simos* appears to be the one that is closer to our initial predictions. Under the assumption of a stage-like lexical access as postulated in Neophytou et al. (2018), it seems that the processing of aspect in formations with **–***simos* takes place at the same timeframe as information related to argument structure. That is, once decomposition has taken place and both morphemes (root and suffix) are activated, information about lexical category of the base takes place during *syntactic licensing.* What follows is the stage of *semantic composition* during which information about argument structure but also about aspect is being processed. Thus, based on findings from **–***simos* we can claim that the processing of aspect belongs to *semantic composition*.

Considering further individual differences among suffixes, we can make the following observations combining both experiments. In pseudowords formed with the suffix *–tos*, low ARs combined with low RTs suggest that AspViols are easily rejected, and aspect is either processed very fast or not at all. This assumption results from the fact that the suffix *–tos* has very weak eventive properties (Markantonatou et al., 1996; Anagnostopoulou, 2003, 2016; Manouilidou, 2006; Anagnostopoulou & Samioti, 2013, 2014) and thus weak aspectual properties, as well. Thus, the low ARs of *–tos* lexical items may indicate the weak, not salient presence of aspect in *–tos* existing deverbal formations, that emerges from the suffix’s lack of eventive properties, and lack of a prototypical Agent argument. Therefore, low RTs arise as the speakers may skip the processing of this type of information, i.e. aspect, saliency of which in the words formed with the suffix *–tos* is as weak as the presence of the event characteristics.

When it comes to pseudowords formed with the suffix *–menos*, results from ARs and RTs align with each other suggesting that aspectual information is not processed as a separate type of information. It is not entirely clear why this is the case. However, the fact that –*menos* appears to also give rise to two different readings, the answer could probably be found in the possibilities of interpretation, in the pseudowords used in the study. That is, it is possible that the specific pseudowords used in the study were interpreted by the participants as only having a Theme argument and a target state interpretation which would entail diminished event properties.

Finally, one last issue we should consider is the morphological dimension of this investigation which might also account for the difference results between Greek and Slovenian. In the current study we used formations with the imperfective stem e.g. *klin-o-ˈtos, which is derived from *ˈklino* ‘to close’ as opposed to the grammatical word *kliˈs-tos* ‘closed’ which is based on the perfective aspect. The perfective aspect is morphologically more salient, and it has a more distinct semantic contribution in Greek, compared to the unmarked imperfective. For AspViols in Slovenian, the perfective aspect was used (Marjanovič et al (2013) and Manouilidou et al (2016)), which is most frequently realized via prefixes, that are easily detectable and decomposable. Hence, Greek speakers possibly found it hard to detect the imperfective aspect in Greek AspViols, which is realized in the middle of the word, and it is occasionally unmarked, compared to the perfective aspect in Slovenian AspViols, which is always marked and realized at the beginning of the word making it easier to be recognized.

To sum up, the present study has provided evidence that aspect might play a role in deverbal word processing in Greek, at least for some suffixes. Comparisons among conditions within the suffix *–simos* at the level of RTs could be suggestive of a step-by-step process where categorial constraints appear to precede the application of both aspectual and argument structure constraints, which apply simultaneously. Hence, if this claim is true, we could argue that after decomposition takes place, the processing of lexical category is carried out during the *licensing* stage, whereas it is possible that the processing of aspect and argument structure follows during *semantic composition*. In this sense, the current study has advanced our understanding of what the *semantic composition* stage could potentially include and it has opened the way for future research on the role of grammatical aspect in deverbal formations. Nevertheless, the inconclusive picture given from the rest of the findings (i.e. the discrepancy observed in the results of Experiment 1 and the results of Experiment 2) regarding the role of aspect in deverbal word processing does not allow us to draw solid claims supporting where aspect stands in speakers’ perception when encountered with the processing of deverbal formations formed with the suffixes *–tos* and *–menos*. Further research is needed with additional suffixes to clarify this issue.

## Appendix: List of AspViols

**Table:** 

AspViols	Transliteration	Translation	Opposite aspect	EXPLAIN	Stem	Transliteration
δινοµένος	ðinoˈmenos	being given	ðοˈzmenos	ˈðin-[imp]+ο	ˈðino	‘to give’
στελµένος	stelˈmenos	being sent	stalˈmenos	ˈsteln-[imp]	ˈstelno	‘to send’
λεγµένος	leγˈmenos	being sent	ipoˈmenos	ˈleγ-[imp]	ˈle(γ)ο	‘to say’
συνδεµένος	sinðeˈmenos	being connected	sinðeðeˈmenos	sinˈðe-[imp]	sinˈðeo	‘to connect’
βλεµµένος	vleˈmenos	being seen	iðoˈmenos	ˈvlep-[imp]	ˈvlepo	‘to see’
διαφθειρµένος	ðiafθirˈmenos	being corrupted	ðiefθarˈmenos	ðiaˈfθir-[imp]	ðiaˈfθiro	‘to corrupt’
κοιταµένος	citaˈmenos	being looked	citaγˈmenos	ciˈt-[imp]	ciˈto	‘to look’
µαθαιµένος	maθeˈmenos	being learnt	maθimenos	maˈθen-[imp]	maˈθeno	‘to learn’
παιρµένος	perˈmenos	being taken	parˈmenos	ˈpern-[imp]	ˈperno	‘to take’
πιµένος	piˈmenos	being drunk	pioˈmenos	ˈpin-[imp]	ˈpino	‘to drink’
πλεµένος	pleˈmenos	being washed	pliˈmenos	ˈplen-[imp]	ˈpleno	‘to wash’
σερνοµένος	sernoˈmenos	being dragged	sirˈmenos	ˈsern-[imp]+ο	ˈserno	‘to drag’
τρωµένος	troˈmenos	being eaten	faγoˈmenos	ˈtro-[imp]	ˈtroo	‘to eat’
φτιαχνοµένος	ftçaxnoˈmenos	being made	ftçaγˈmenos	ˈftiaxn-[imp]+ο	ˈftiaxno	‘to make’
αρρωσταιµένος	arosteˈmenos	being sickened	arostiˈmenos	aroˈsten-[imp]	aroˈsteno	‘to get sick’
κλαιµένος	kleˈmenos	being cried	klaˈmenos	ˈkle-[imp]	ˈkleo	‘to cry’
καιµένος	keˈmenos	being burnt	kaˈmenos	ˈke-[imp]	ˈkeo	‘to burn’
κλειµένος	kliˈmenos	being closed	kliˈzmenos	ˈklin-[imp]	ˈklino	‘to close’
ζεσταιµένος	zesteˈmenos	being warmed	zestaˈmenos	zeˈsten-[imp]	zeˈsteno	‘to warm’
περνοµένος	pernoˈmenos	being passed	peraˈzmenos	perˈn-[imp]+ο	perˈno	‘to pass’
διαδιδόσιµος	ðiaðiˈðosimos	being spread	ðiaˈðosimos	ðiaˈðið-[imp]+ο	ðiaˈðiðo	‘to spread’
συγκρινόσιµος	sigriˈnosimos	being compareable	sigriˈnosimos	sigriˈn-[imp]+ο	sigriˈno	‘to compare’
αναγνωριζόσιµος	anaγnoriˈzosimos	being recognizeable	anaγnoˈrisimos	anaγnoˈriz-[imp]+ο	anaγnoˈrizo	‘to recognize’
εντοπιζόσιµος	edopiˈzosimos	being detectable	edoˈpisimos	edoˈpiz-[imp]+ο	edoˈpizo	‘to detect’
συνεργαζόσιµος	sinerγaˈzosimos	being cooperateable	sinerˈγasimos	sinerˈγaz-[imp] + o	sinerˈγazome	‘to cooperate’
αναπαραγώσιµος	anaparaˈγosimos	being reproduceable	anapaˈraksimos	anapaˈraγ-[imp]+ο	anapaˈraγo	‘to reproduce’
ανιχνευόσιµος	anixneˈvosimos	being traceable	aniˈxnefsimos	aniˈxnev-[imp]+ο	aniˈxnevo	‘to trace’
αρδευόσιµος	arðeˈvosimos	being irrigateable	arˈðefismos	arˈðev-[imp]+ο	arˈðevo	‘to irrigate’
εκλεγόσιµος	ekleˈγosimos	being electable	eˈkleksimos	eˈkleγ-[imp]+ο	eˈkleγo	‘to elect’
εκπαιδευόσιµος	ekpeðeˈvosimos	being trainable	ekpeˈðefsimos	ekpeˈðev-[imp]+ο	ekpeˈðevo	‘to train’
εξαγοραζόσιµος	eksaγoraˈzosimos	being buyable off	eksaγoˈrasimos	eksaγοˈraz-[imp]+ο	eksaγοˈrazo	‘to buy off’
επιλεγόσιµος	epileˈγosimos	being chooseable	epiˈleksimos	epiˈleγ-[imp]+ο	epiˈleγo	‘to choose’
θεραπευόσιµος	θerapeˈvosimos	being cureable	θeraˈpefismos	θeraˈpev-[imp]+ο	θeraˈpevo	‘to cure’
κολαζόσιµος	kolaˈzosimos	being punishable	koˈlasimos	koˈlaz-[imp]+ο	koˈlazo	‘to punish’
πινόσιµος	piˈnosimos	being drinkable	ˈposimos	ˈpin-[imp]+ο	ˈpino	‘to drink’
προσδιοριζόσιµος	prozðioriˈzosimos	being defineable	prozðioˈrisimos	prozðδioˈriz-[imp]+ο	prozðδioˈrizo	‘to define’
τρώσιµος	ˈtrosimos	being eatable	faˈγosimos	ˈtro-[imp]	ˈtroo	‘to eat’
εργαζόσιµος	erγaˈzosimos	being workable	erˈγasimos	erˈγaz-[imp]+ο	erˈγazome	‘to work’
µεταβιβαζόσιµος	metavivaˈzosimos	being transferable	metaviˈvasimos	metaviˈvaz-[imp]+ο	metaviˈvazo	‘to transfer’
προσβαινόσιµος	prozveˈnosimos	being accessible	proˈzvasimos	proˈzven-[imp]+ο	proˈzveno	‘to access’
γραφοτός	γrafoˈtos	being writeable	γraˈptos	ˈγraf-[imp]+ο	ˈγraf	‘to write’
λεγοτός	leγoˈtos	being sayable	riˈtos	ˈleγ-[imp]+ο	ˈleγo	‘to say’
ριχνοτός	rixnoˈtos	being throwable	riˈxtos	ˈrixn-[imp]+ο	ˈrixno	‘to throw’
ακουτός	akuˈtos	being listenable	akuˈstos	aˈku-[imp]	aˈkuo	‘to listen’
ανεχοτός	anexoˈtos	being tolerateable	aneˈktos	aˈnex-[imp]+ο	aˈnexome	‘to tolerate’
δενοτός	ðenoˈtos	being tieable	ðeˈtos	ˈðen-[imp]+ο	ˈðeno	‘to tie’
εκλεγοτός	ekleγoˈtos	being electable	ekleˈktos	eˈkleγ-[imp]+ο	eˈkleγo	‘to elect’
επιλεγοτός	epileγoˈtos	being chooseable	eˈpilektos	epiˈleγ-[imp]+ο	epiˈleγo	‘to choose’
παιρνοτός	pernoˈtos	being takeable	parˈtos	ˈpern-[imp]+ο	ˈperno	‘to take’
σερνοτός	sernoˈtos	being dragable	sirˈtos	ˈsern-[imp]+ο	ˈserno	‘to drag’
στρωνοτός	stronoˈtos	being layable	stroˈtos	ˈstron-[imp]+ο	ˈstrono	‘to lay’
σφιγγοτός	sfigoˈtos	being tightenable	sfiˈxtos	ˈsfig-[imp]+ο	ˈsfigo	‘to tighten’
τυλιγοτός	tiliγoˈtos	being wrapeable	tiliˈxtos	tiˈliγ-[imp]+ο	tiˈliγo	‘to wrap’
φθειρτός	fθirˈtos	being wearable out	fθarˈtos	ˈfθir-[imp]	ˈfθiro	‘to wear out’
φωναζοτός	fonazoˈtos	being shoutable	fonaˈxtos	foˈnaz-[imp] + o	foˈnazo	‘to shout’
τσιριζοτός	tsirizoˈtos	being screamable	tsiriˈxtos	tsiˈriz-[imp] + o	tsiˈrizo	‘to scream’
υπαρχοτός	iparxoˈtos	being existable	iparˈktos	iˈparx-[imp] + o	iˈparxo	‘to exist’
ανοιγοτός	aniγoˈtos	being openable	aniˈxtos	aˈniγ-[imp] + o	aˈniγo	‘to open’
κλεινοτός	klinoˈtos	being closeable	kliˈstos	ˈklin-[imp] + o	ˈklino	‘to close’

## Data Availability

The data that support the findings of this study are available from the corresponding author upon request.

## Footnotes

1 Given that the focus of the paper is on Greek language, we will not expand on the description of grammatical aspect in Slovenian.

2 As the following examples illustrate a by-phrase is not possible with –*tos* formations when they have a characteristic state reading: ** To kuˈti ˈitan aniˈx-to me kliˈði.* ‘The box was opened with a key’ **Ta* *makaˈronia ˈitan vraˈsta apo to ˈJani* ‘The spaghetti was boiled by Jiannis. (Markantonatou et al., 1996; Anagnostopoulou, 2003, 2016; Manouilidou, 2006; Anagnostopoulou & Samioti, 2013, 2014); Anagnostopoulou, 2016). However, –*tos* formations in their “ability/possibility” reading license Agent and Instrument PPs *I* *isto΄ria tu΄ine piste΄fti a΄po΄olus‘* his story can be believed by everyone’, *to vu΄no ΄ine ora΄to me ta ΄kjalja ‘*The mountain is visible with binoculars’ (Anagnostopoulou & Samioti, 2014). For the syntactic analysis of this, the reader is referred to Anagnostopoulou & Samioti (2014) and Samioti (2015).

3 Anagnostopoulou (2003) studying the syntactic properties of *–menos* revealed a dual attachment, that is –*menos* can either be attached high to VP (VoicePhrase) to create a "result state" nominal, licensing an agentive by-phrase, e.g., *Ta makaˈroɲa ˈine vrazmena aˈpo to ˈJani*. ‘Spaghetti is boiled by John’, or low VP (verbalPhrase) to create a "target state", not licensing an agentive by-phrase, e.g. *I ˈporta ˈine kliˈzmeni (*aˈpo to ˈJani).* ‘The door is closed (*by John)’.

4 The following psycholinguistic variables were used: stem frequency, number of item’s phonological neighbours, number of item’s orthographic neighbours, number of stem’s phonological neighbours, number of stem’s orthographic neighbours, phonological cohort, orthographic cohort, item’s length, stem’s length.
